# Supplementary material for: Rationale for Combining the BCL2 Inhibitor Venetoclax with the PI3K Inhibitor Bimiralisib in the Treatment of IDH2- and FLT3-Mutated Acute Myeloid Leukemia
Source: Int J Mol Sci. 2022 Oct 20;23(20):12587. doi: 10.3390/ijms232012587 (PMC9604078; doi:10.3390/ijms232012587)
Supplement: Supplementary file 1 [file ijms-23-12587-s001.zip › ijms-1916794-supplementary.pdf]

Supplementary materials

Rationale for combining the BCL2 inhibitor venetoclax with the PI3K inhibitor bimiralisib in the treatment of IDH2- and FLT3- mutated acute myeloid leukemia

Katja Seipel [1*], Yvo Brügger[1] , Harpreet Mandhair [1], Ulrike Bacher[2] and Thomas Pabst [3,*]

1. Affiliation 1; Department for Biomedical Research (DBMR), University of Bern, 2008 Bern, Switzerland
2. Affiliation 2: Department of Hematology, University Hospital Bern, 3010 Bern, Switzerland
3. Affiliation 3; Department of Medical Oncology, University Hospital Bern, 3010 Bern, Switzerland

* Correspondence: katja.seipel@dbmr.unibe.ch; Tel.: +41 31 6320934

Supplementary Materials

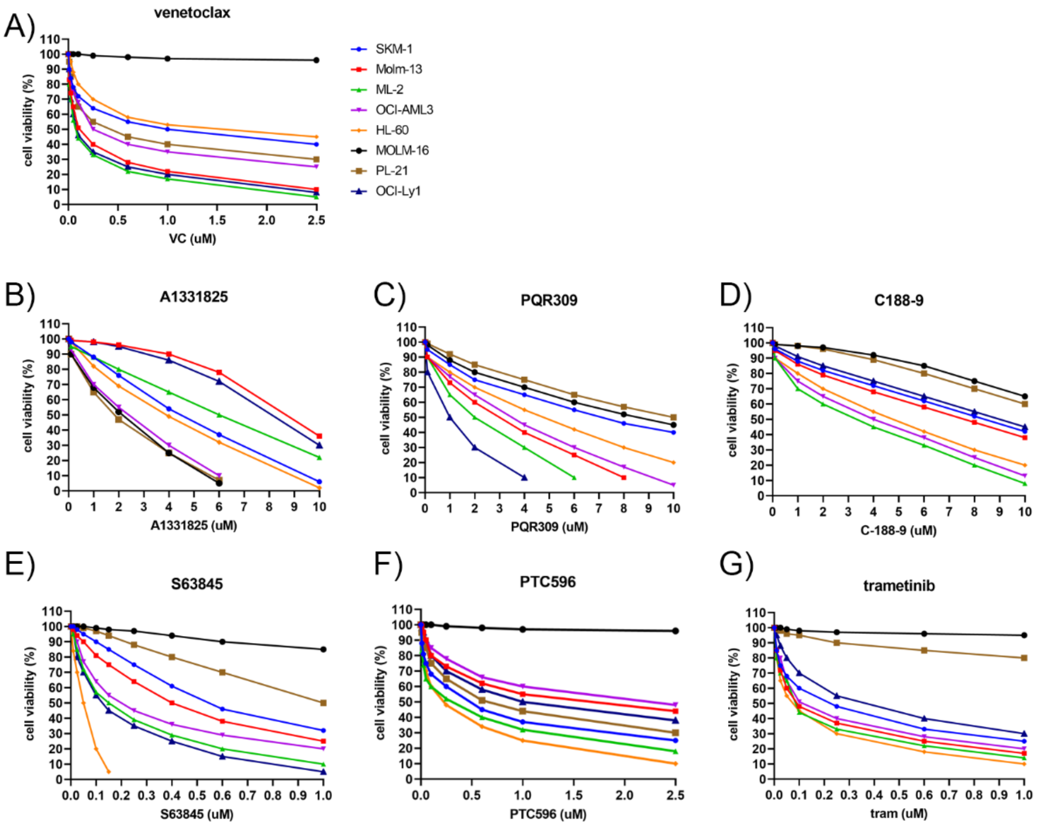

Figure S1. Dose response of AML cell lines treated with venetoclax (A), A1331825 (B), PQR309 (C), C-188-9 (D), S63845 (E), PTC596 (F), trametinib (G). Cell viability data are average values of multiple repeat measurements per dosage. Standard deviation 5–10%.

Table S1. IC50 values cell lines (μM).

	targeted therapy						
cell line	**venetoclax**	**A1331825**	**PQR-309**	**C-188-9**	**PTC596**	**S63845**	**trametinib**
**target**	**BCL-2**	**BCL-XL**	**PI3K, mTOR**	**STAT3**	**BMI-1**	**MCL-1**	**MEK**
HL-60	1	4	5	5	0.2	0.1	0.08
ML-2	0.08	2	3	4	1.5	0.5	0.12
MOLM-13	0.1	6	2	4	0.3	0.01	0.12
MOLM-16	>10	2	10	>10	1.1	10	10
OCI-AML3	0.2	4	10	8	0.5	0.2	0.1
PL-21	10	2	10	>10	0.8	1	10
SKM-1	2	8	3	8	1.2	0.5	0.12
OCI-Ly1	0.06	8	1	8	1	0.12	0.3

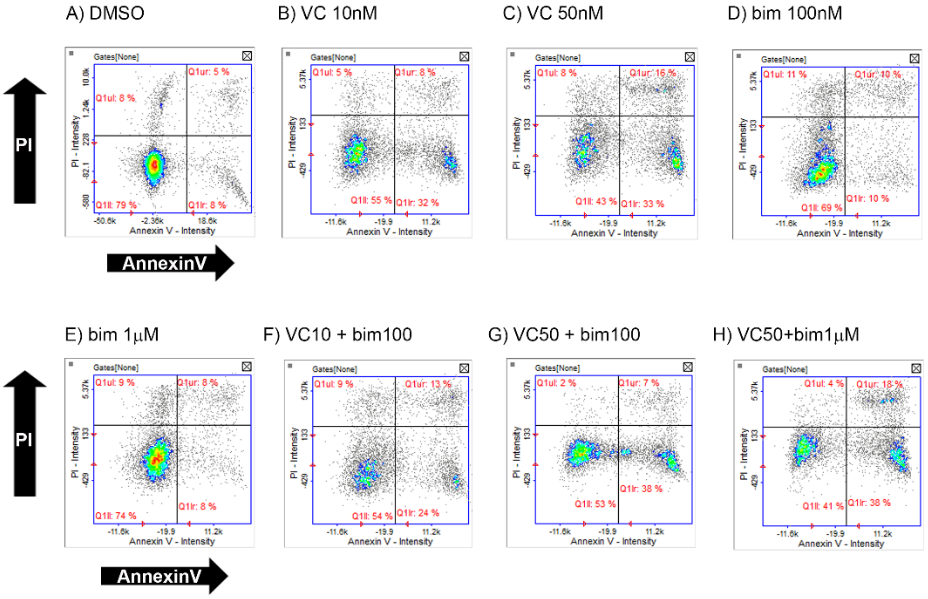

Figure S2. Imaging cytometry in MOLM-13 cells treated for 20 hrs with venetoclax (VC) and bimiralisib (bim). A) mock-treated (DMSO), B) VC 10 nM, C) VC 50 nM, D) bim 100 nM E) bim 1 µM, F) VC 10 nM and bim 100 nM, G) VC 50 nM and bim 100 nM, H) VC 50 nM and bim 1 µM, depicting induction of apoptosis and cell death using annexinV and PI staining. Interpretation: Ann lo, PI lo (lower left quadrant): vital cells; Ann hi, PI lo (lower right quadrant): early apoptotic cells; Ann hi PI hi (upper right quadrant): late apoptotic cells; Ann lo, PI hi (upper left quadrant): necrotic cells.

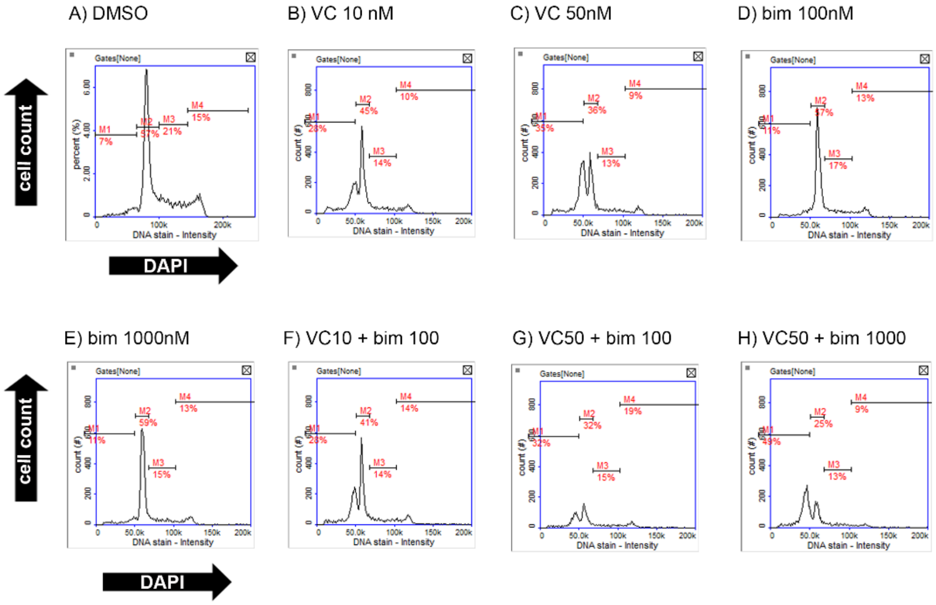

Figure S3. Cell cycle analysis in MOLM-13 cells treated for 20 hrs with venetoclax (VC) and bimiralisib (bim). A) mock-treated (DMSO), B) VC 10 nM, C) VC 50 nM, D) bim 100 nM E) bim 1 µM, F) VC 10 nM and bim 100 nM, G) VC 50 nM and bim 100 nM, H) VC 50 nM and bim 1 µM, depicting cell cycle phases using DAPI staining. Depending on DAPI stain-ing intensity cells were classified as subG1 (M1), G0/G1 (M2), S phase (M3) or G2 phase (M4).

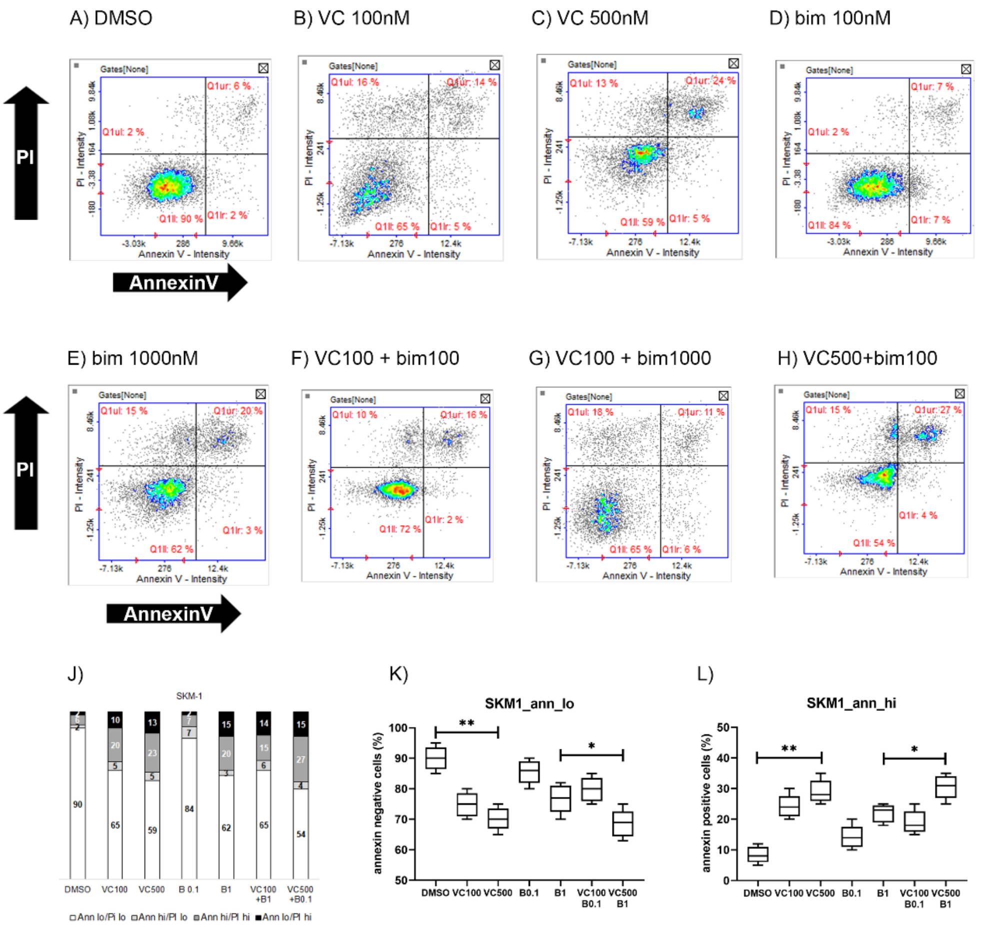

Figure S4. Imaging cytometry in SKM-1 cells treated for 20 hrs with venetoclax (VC) and bimiralisib (bim). A) mock-treated (DMSO), B) VC 100 nM, C) VC 500 nM, D) bim 100 nM E) bim 1 µM, F) VC 100 nM and bim 100 nM, G) VC 50 nM and bim 100 nM, H) VC 50 nM and bim 1 µM, depicting induction of apoptosis and cell death using annexinV and PI staining. Depending on Annexin V and PI staining intensity cells were classified as vital (Ann lo, PI lo), early apoptotic (Ann hi, PI lo), late apoptotic (Ann hi, PI hi) or necrotic (Ann lo, PI hi) (I). Treatment induced loss of viable cells (J) and treatment increase in apoptotic cells (K) were significantly enhanced in combination treatments. Significance of differences in median values was calculated by Mann-Whitney test. Significance denoted for $p < 0.05$ (*); $p < 0.005$ (**); no significance denoted for $p > 0.05$.

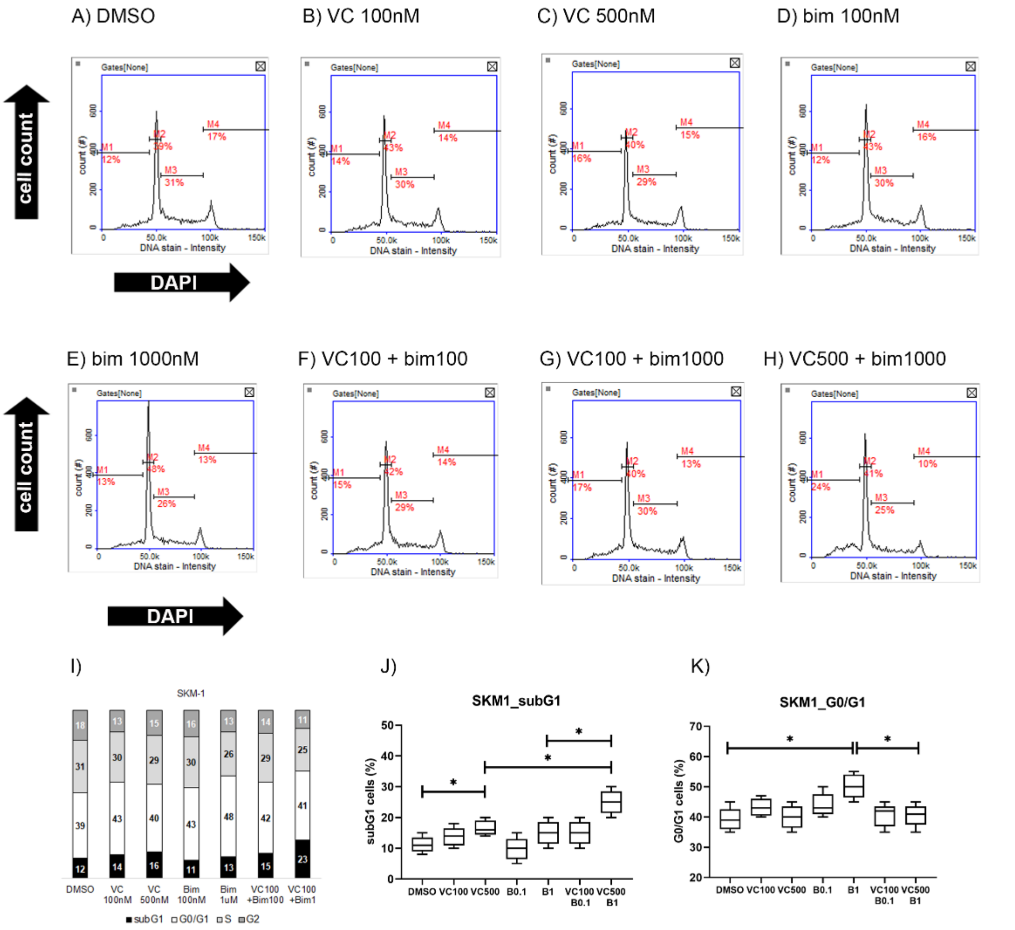

Figure S5. Imaging cytometry in SKM-1 cells treated for 20 hrs with venetoclax (VC) and bimiralisib (bim). A) mock-treated (DMSO), B) VC 100 nM, C) VC 500 nM, D) bim 100 nM E) bim 1 µM, F) VC 100 nM and bim 100 nM, G) VC 50 nM and bim 100 nM, H) VC 50 nM and bim 1 µM, depicting induction of cell cycle arrest and cell death (subG1 fraction) using DAPI staining. Depending on DAPI staining intensity cells were classified as subG1 (M1), G0/G1 (M2), S phase (M3) or G2 phase (M4) (I). Treatment induced cell death (subG1 frac-tion) (J) was significantly enhanced in combination treatment. Bimiralisib induced G1 cell cycle arrest (K). Significance of differences in median values was calculated by Mann-Whitney test. Significance denoted for $p < 0.05$ (*); no significance denoted for $p > 0.05$.

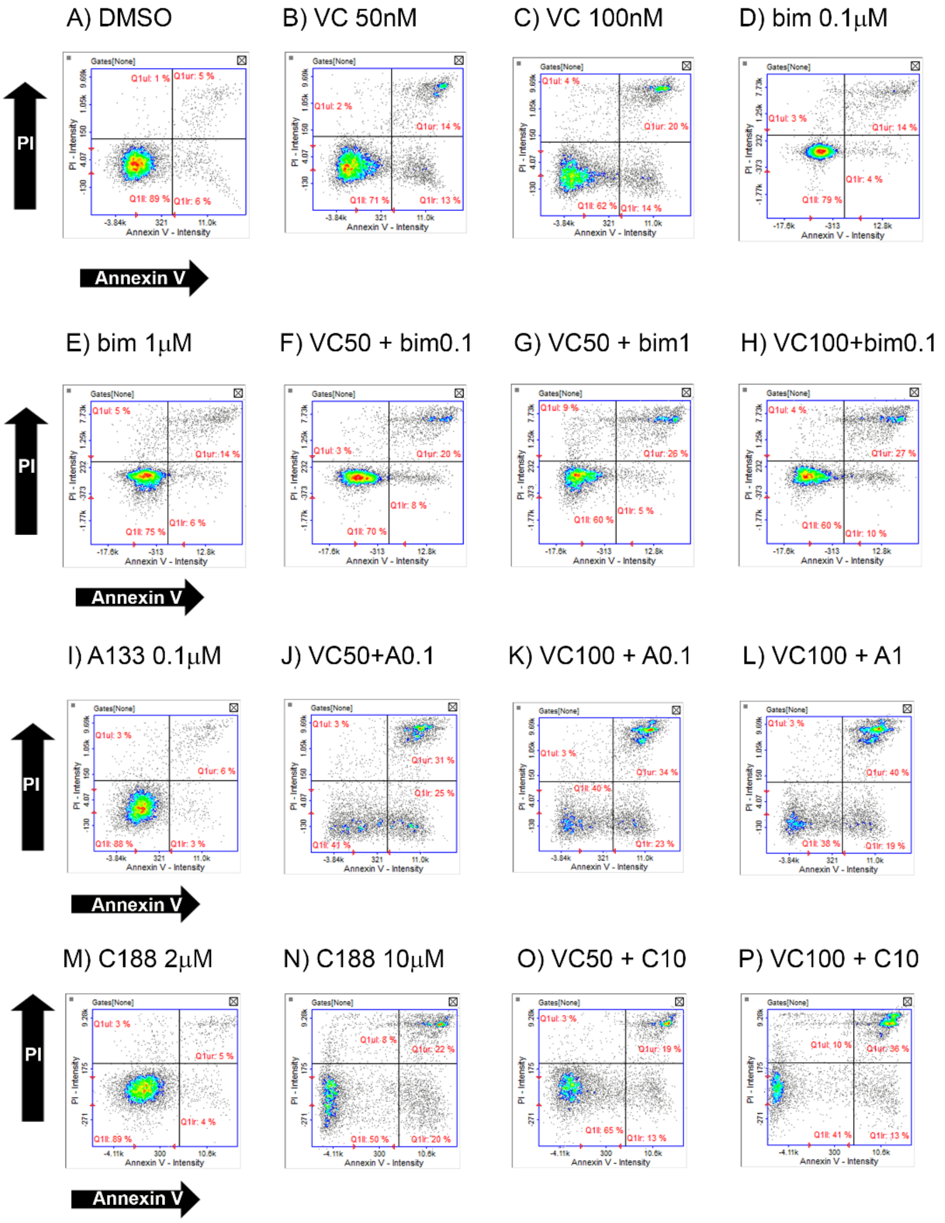

Figure S6. Imaging cytometry in ML-2 cells treated for 20 hrs with venetoclax (VC) and bimi-ralisib (bim), A1331825 or C-188-9. A) mock-treated (DMSO), B) VC 50 nM, C) VC 100 nM, D) bim 100 nM E) bim 1 µM, F) VC 50 nM and bim 100 nM, G) VC 50 nM and bim 1 µM, H) VC 100 nM and bim 100 nM, I) A1331825 0.1 µM, J) A1331825 1 µM, K) VC 50 nM and A 0.1µM, L) VC 100 nM and A 0.1 µM, M) C188-9 2 µM, N) C188-9 10 µM, O) VC 50 nM and C 10 µM, P) VC 100 nM and C 10 µM, depicting induction of apoptosis and cell death using annexinV and PI staining. Depending on Annexin V and PI staining intensity cells were classified as vital (Ann lo, PI lo), early apoptotic (Ann hi, PI lo), late apoptotic (Ann hi, PI hi) or necrotic (Ann lo, PI hi).

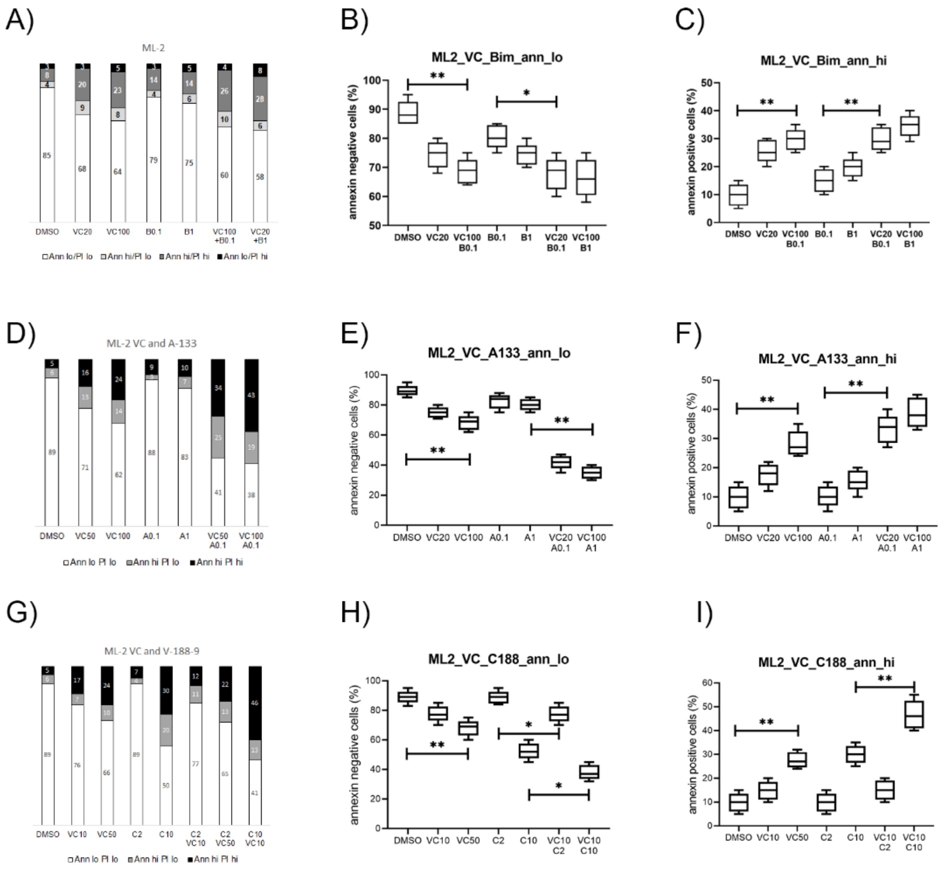

Figure S7. Induction of apoptosis and cell death in ML-2 cells treated for 20 hrs with veneto-clax (VC) and bimiralisib (A,B,C), A1331825 (D,E,F) or C-188-9 (G,H,I). Depending on An-nexin V and PI staining intensity cells were classified as vital (Ann lo, PI lo), early apop-totic (Ann hi, PI lo), late apoptotic (Ann hi, PI hi) or necrotic (Ann lo, PI hi) (I). Treatment induced loss of vital cells (B,E,H) and treatment induced increase in apoptotic cells (C,F,I) were significantly enhanced in combination treatment. Significance of differences in me-dian values was calculated by Mann-Whitney test. Significance denoted for p < 0.05 (*); p < 0.005 (**); no significance denoted for p > 0.05.

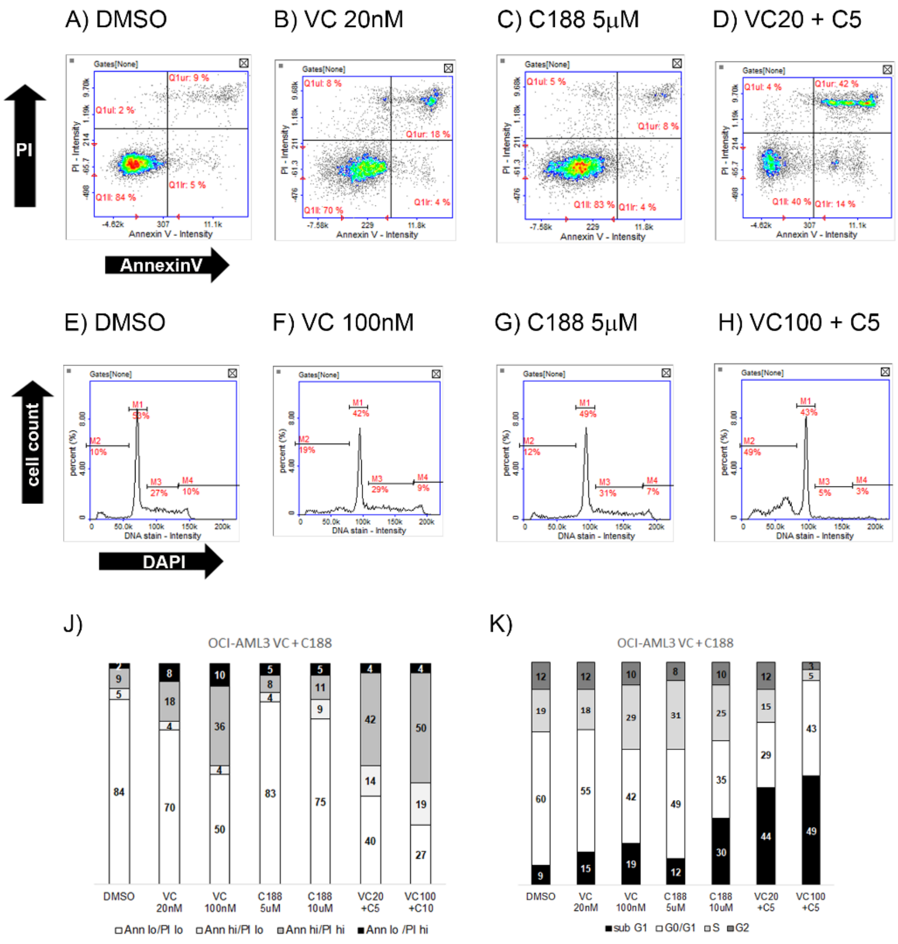

Figure S8. Induction of apoptosis and cell death in OCI-AML3 cells treated for 20 hrs with venetoclax (VC) and C-188-9. Induction of apoptosis and cell death using annexinV and PI staining in A) mock-treated (DMSO), B) VC 20 nM, C) C188-9 5 µM, D) VC 20 nM and C188 5 µM treated cells. Induction of cell cycle arrest and cell death (subG1 fraction) using DAPI staining in E) mock-treated (DMSO), F) VC 100 nM, G) C188-9 5 µM, H) VC 100 nM and C188 5 µM. Depending on Annexin V and PI staining intensity cells were classified as vital (Ann lo, PI lo), early apoptotic (Ann hi, PI lo), late apoptotic (Ann hi, PI hi) or necrotic (Ann lo, PI hi) (J). Depending on DAPI staining intensity cells were classified as subG1, G0/G1, S phase or G2 phase (K).

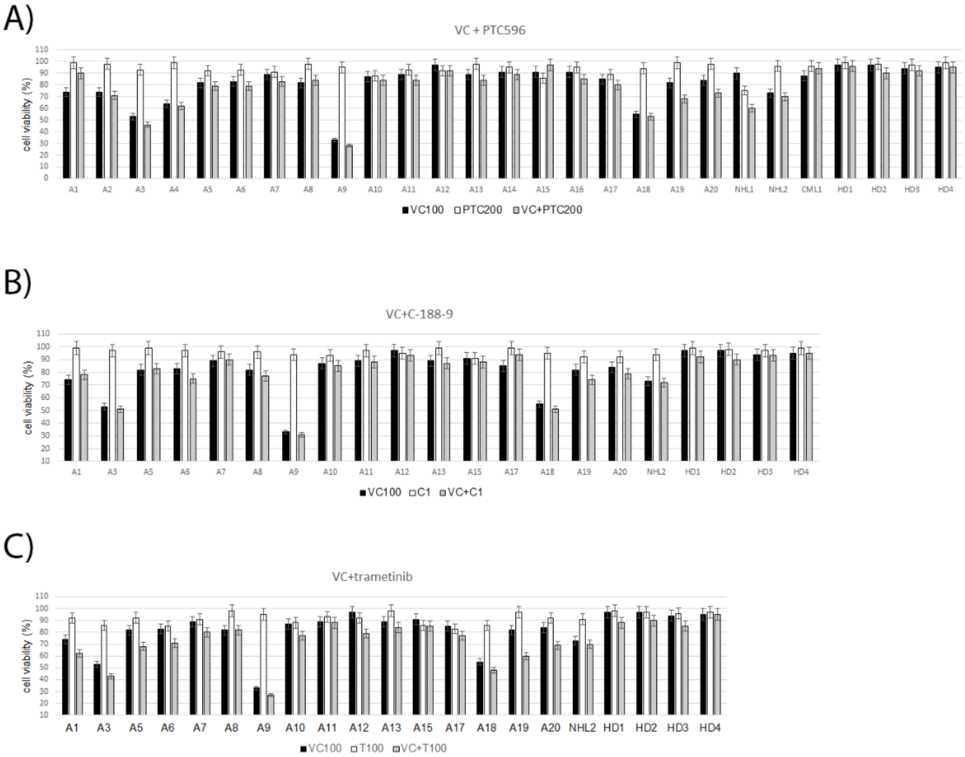

Figure S9. Hematological cells in vitro response to venetoclax and various combination treat-ments. Cell viability was determined in mononuclear cells isolated from AML patients or healthy donors (HD) peripheral blood or bone marrow after 20 hour treatment with 100 nM venetoclax and 200 nM PTC596 (A), 1 μM C-188-9 (B), or 100 nM trametinib (C).
